# Photolytic Behavior of Silver Iodide^1^

**DOI:** 10.6028/jres.067A.032

**Published:** 1963-08-01

**Authors:** G. Burley

## Abstract

Silver iodide exposed to high intensity radiation in the visible light spectrum was found to yield a powder X-ray diffraction pattern showing marked deviations from ideality. It was found possible to correlate these with a decrease in primary extinction, indicating a constant progress from an ideal to a mosaic type crystallinity. Large single crystals showed pronounced asterism in transmission Laue photographs under similar experimental conditions. Small amounts of colloidal silver were detected. A mechanism for this process in silver iodide is proposed, in general agreement with the theory of the photographic process. The primary difference from the other silver halides appears to be a considerably slower rate, permitting the observation of a two step process in detail.

## 1. Introduction

No detailed investigation of the mechanism of the changes induced in silver iodide by exposure to light in the visible region appears to have been made previously. In the course of this work, the photoytic behavior of silver iodide has been found to differ considerably from that of the other silver halides. In general, polycrystalline specimens of both silver chloride and silver bromide darken rapidly under these conditions, due to the formation of free silver. Powder X-ray diffraction patterns clearly show a decrease of the pattern for the halide, accompanied by a corresponding increase of that for silver, with increasing length of exposure to a constant intensity light source. The behavior of silver iodide under similar conditions and an analysis of the results obtained constitute the results reported in this paper.

## 2. Experimental Procedure

Single crystals of silver iodide were exposed to the light of an arc lamp for 1 week. Initially transparent, they slowly became opaque. Microscopic examination showed that the initially smooth surfaces gradually roughened and resembled a polycrystalline aggregate. This alteration gradually progressed into the interior of the crystals. Certain sensitizers and halogen acceptors, such as sulfur dioxide and *n*-butyl alcohol, were found to accelerate this process.

Because the action of light on silver iodide appeared to be primarily a surface phenomenon most subsequent work was done with powder specimens.

The powder was packed into a rectangular cavity and the surface smoothed. The mount was placed in a closed container and irradiated with a bar filament microscope lamp through a 5 mil polyester sheet. An atmosphere of *n*-butyl alcohol was maintained at its vapor pressure. This was chosen because it accelerated the reaction only to such an extent as not to hide its essential details. The sample was irradiated for a fixed length of time and an X-ray diffractometer pattern obtained immediately. An angular range sufficient to cover the first nine lines of the diffraction pattern for the hexagonal phase was traversed each time. For the copper X-radiation used this included the range from 20 to 50° in 2*θ*, where *θ* is the Bragg angle. A scanning rate of 0.5° per minute of the angle 20 was used for maximum resolution. Approximately monochromatic radiation was obtained by use of a nickel foil filter. Cumulative exposure times to light of 200 hr were reached in this manner.

In a related experiment a large hexagonal single crystal, of approximately 5 mm diam and 1 mm thickness, prepared by slow evaporation of a 50 percent aqueous solution of hydriodic acid saturated with silver iodide, was exposed to light in the same manner. Laue transmission X-ray diffraction diagrams were taken at timed intervals. Molybdenum X-radiation was used to minimize absorption effects.

## 3. Results

**Figure 1:**
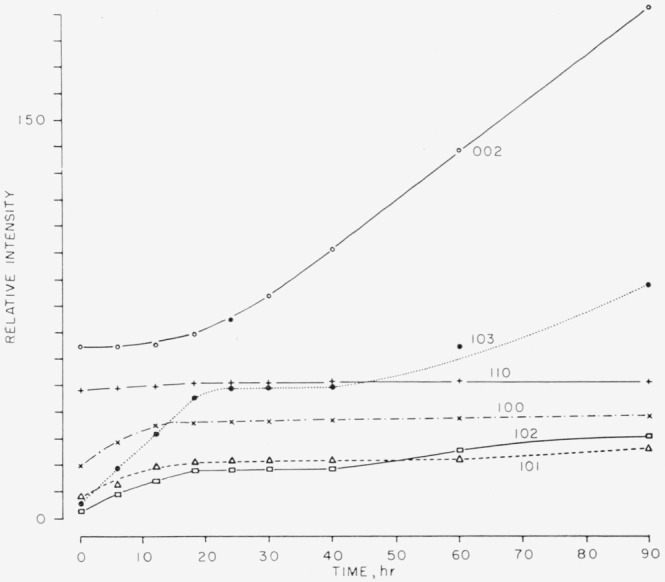
Relative intensities of several low angle powder X-ray diffraction lines for silver iodide, as a function of irradiation time by light in the visible region (*CuK*α radiation).

The powder X-ray diffraction patterns for silver iodide showed large changes in the intensities of certain lines, related to the length of exposure to light. The relative intensities of certain lines as a function of cumulative exposure time are shown in figure 1. The most striking feature of this graph is the fact that there was a rapid and immediate increase for the (100), (101); (102), and (103) reflections, while other reflection intensities remained constant. After a period of time, varying with the light intensity and spectral characteristics of the source, these reflections reached a plateau. After this induction period the (002) reflection intensity then began to increase while the others leveled off. There was no decrease in intensity for any reflection.

Secondly, the number and angular positions of the X-ray powder diffraction lines were found to remain essentially invariant during the entire procedure. No systematic shift was detectable.

Thirdly, the powder diffraction patterns of light-irradiated silver iodide were re-examined for the presence of metallic silver. The (110) reflection of silver has the largest intensity and appears at a 2*θ* angle of 38.11°. On the diffraction patterns of silver iodide samples irradiated for 50 hr a very small and broad peak was located at this position. The peak increased slightly in height with successive exposures to light. This clearly established the presence of finely divided silver in amounts large enough to be detected by the X-ray method after exposure times longer than 50 hr.

## 4. Intensity Changes

The problems to be solved included finding an explanation for the anomalous intensity changes observed and correlating this with the theory of the photographic process.

**Figure 2:**
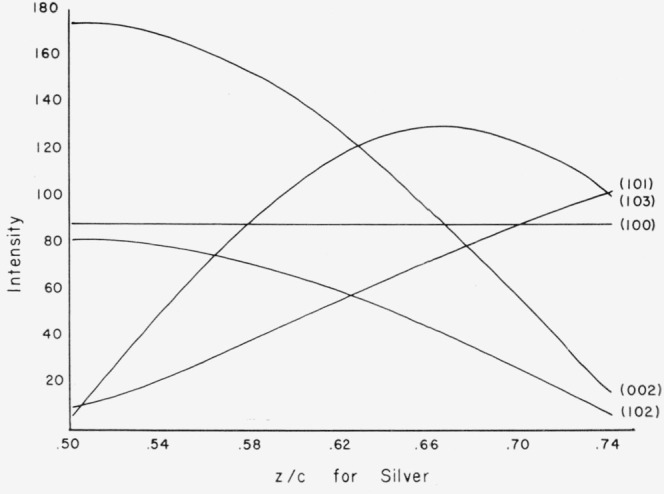
Calculated intensities for several reflections of hexagonal silver iodide, as a function of the variable z/c positional parameter for the silver atom.

The effect of varying the positional parameters of the atoms on the magnitude of the intensities was investigated first. Silver iodide can exist in both a cubic zincblende and a hexagonal wurtzite form at room temperature. No distortion of the structure for the cubic zincblende-type phase is possible without destroying the symmetry. In the hexagonal wurtzite-type structure the *x* and *y* parameters of both atoms are fixed and only the *z* parameters can vary. In addition, the origin may be chosen arbitrarily and can be placed at the iodine position for convenience. The only variable possible for an atomic shift is then the *z* parameter of the silver atom. The variation of the amplitude of the structure factors for the observed diffraction lines was calculated over a fairly large range of values for the silver *z* parameter. The result is shown in graphical form in figure 2. It is obvious that no atomic shift is possible which will increase all intensities simultaneously.

Another possibility which had to be considered was the existence of stacking faults. This refers to random deviations of layers from the required progression for either the hexagonal or face-centered cubic lattice which are ABAB … and ABCABC …, respectively. Deviations may be introduced by either growth or deformation faulting.

The experimental details for this behavior have been reported in the closely analogous case of cobalt metal [1].^3^ The general theory for mistakes in layer structures was published at the same time by Wilson [2, 3]. The diffraction pattern for a layer type structure with stacking faults has sharp diffraction lines when (*h–k*)/3 has integer values (where *h* and *k* are Miller indices); in all other cases the lines are broadened. This behavior was not observed for silver iodide.

The lines with integer values of (*h–k*)/3, indexed in the hexagonal system, are, however, the only ones which occur also for the zincblende-type cubic polymorph. These were the only diffraction lines whose intensity did not change during the initial phase of the illumination. The implications of this behavior will be considered later.

An explanation in terms of a detect structure appears equally unlikely since the line widths and incoherent background scattering did not change markedly. Annealing of a defect structure does increase the peak heights of the diffraction diagram, but the area under the peak profiles remains constant. Since the line width variations observed in this experiment were within the experimental error, none of the peak height changes can be attributed to this cause.

Radical intensity differences for lines in an X-ray powder diffraction diagram may also be due to preferred orientation of the crystallites. For layer structure especially one, or several, sets of planes may have more than a proportional share of these crystallites in reflecting position. In this case certain diffraction peaks, originating from sets of planes having a larger than statistical number in reflecting position are enhanced, and the intensity from most other sets of planes is decreased. This was not observed for silver iodide.

A closely related effect concerns a change of relative intensities caused by crystal growth or aggregation on a submicroscopic scale in certain preferred directions. Since two distinct stages in the change were observed, during each of which certain diffraction lines increased in height while none diminished it seems unreasonable to suppose that under these circumstances a change in preferred orientation or crystal habit occurred.

The only major remaining cause for large intensity changes was the possibility of extinction in the initial samples. This refers to the attenuation of the diffracted beam by regions of perfection within the crystallite. Since there appeared to be a surface fragmentation of the larger single crystals by light it was easy to extend this idea to the small crystallites in the powder. This fragmentation could be expected to reduce the coherently scattering block size and consequently to decrease the extinction. The details of this mechanism will be discussed in the following section.

**Figure 3:**
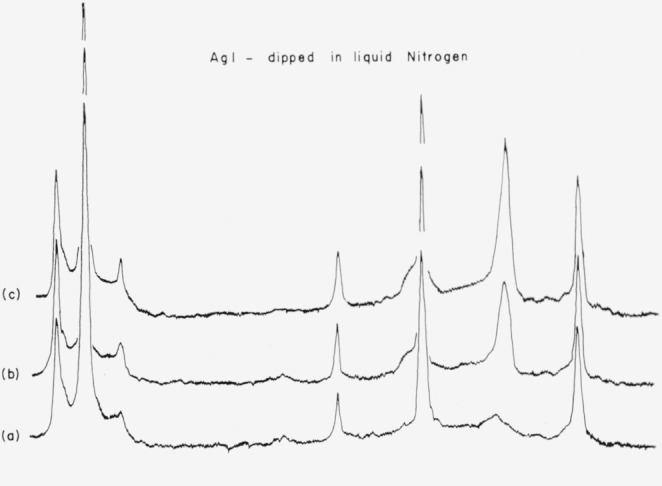
Powder X-ray diffraction profiles for silver iodide. Initial.3 rain immersion in liquid nitrogen.10 min immersion in liquid nitrogen.

A generally used method of reducing the perfection of a crystal depends on the use of thermal shock to introduce mosaic-type disorder. The powder sample of silver iodide was immersed in liquid nitrogen repeatedly and X-ray diffraction patterns were obtained. The intensities of the diffraction lines changed in proportionately the same amounts to the first plateau stage as for the samples irradiated by light, as shown in figure 3. This constituted another indication that the observed intensity changes were due to a decrease in extinction and led to the more detailed study described below.

## 5. Extinction

Darwin [4] in 1922 derived an approximation by which allowance can be made for the primary extinction in reflections from crystals whose units are larger than ideally mosaic. The ratio of the diffracted intensity with extinction to that without extinction is given by

$$\begin{array}{l} \frac{I_{\text{ext}}}{I_{0}}=\frac{\tanh\left(sq\right)}{sq}\\ \;=1—\left\{\frac{4k}{3}{\;}^{2}{\left(\frac{e^{2}}{mc^{2}}\right)}^{2}pF^{2}t_{0}^{2}/\gamma_{0}\gamma_{r}\right\} \end{array}$$

where

*s*: = number of reflecting planes in perfect sequence,
*q*: = amplitude reflected from a single plane of atoms,
*N*: = Avogadro’s number,
*d*: = interplanar spacing,
$\frac{e^{2}}{mc^{2}}$: = classicall radius of electron=2.818×10^−13^ cm,
*p*: = polarization factor,
*F*: = structure factor,
*t*_0_: = thickness penetrated,
*γ*_0_: *γ_r_*, = direction cosines with the normal to the plate for the incident and diffracted rays.

The factor 4*k*/3 has an approximate numerical value of 1/2. The term enclosed by the braces is the extinction coefficient and will hereafter be designated *ϵ*. This expression is essentially wavelength independent for any one reflection. Darwin based his original expression on the assumptions of a plate of infinite lateral extent with the reflecting planes parallel to the surface.

Neither of Darwin’s assumptions actually holds for a poly crystalline sample. For this the scattering regions are of approximately equal dimensions and the planes are at arbitrary angles to the surface.

In 1951 Ekstein derived an independent extinction formula on the basis of small spherical perfect crystallites [5]. It was originally derived for neutron diffraction and involves peak rather than integrated intensities. For electromagnetic radiation it has the form

$$\frac{I_{\text{ext}}}{I_{0}}=1—\left\{\frac{7}{16}{\left(\frac{e^{2}}{mc^{2}}\right)}^{2}N^{2}F^{2}D^{2}\lambda^{2}\right\}.$$

This formula has a second order wave length dependence as shown here. Thus it should be possible experimentally to confirm the applicability of one or the other formula.

Since at least a substantial part of the observed intensity anomaly for unirradiated silver iodide powder could be ascribed to the primary extinction effect, the wavelength dependence could be investigated. For this purpose manganese filtered iron, nickel filtered copper, and zirconium filtered molybdenum X-radiations were used in three separate series of experiments. The wavelengths of these are 1.9360 A, 1.5405 A, and 0.7093 A, respectively.

The results obtained for the (100), (101), (102), and (103) reflections are shown in table 1. The peak intensities were used here. Because of the uniformity of the line width this is directly proportional to the integrated area under each curve. A polarization correction, designated as *p*, was applied. *I*_init_ represents the intensity with an extinction effect and *I*_final_ the intensity without this effect; the ratio of *I*_init_/*pI*_final_ remains approximately constant for all three wavelengths used. Thus the experimental data indicate approximately a zero order wavelength dependence, in agreement with the Darwin formula.

Most of the crystallites grow in the form of either plates or truncated hexagonal pyramids. The dominant face is thus the basal plane. The reference vector for the direction cosines was taken as the normal to this plane. Using the Darwin formula the relative extinction coefficients were calculated for the {10*l*} reflections for each of the three wave lengths used. Values for the structure factor, *F*, were obtained from single crystal precession photographs. The results are shown in table 2. Since only relative values of the *F*^2^ terms were used, these were not directly comparable to the observed values. In the absence of any other criteria the (103) reflection was used as a comparison standard in each case. The significance of the agreement is thus primarily in the value of *ϵ* obtained for the other reflections. The agreement between the observed and calculated values is quite good and can be taken as final confirmation that the initial increases in intensity are due to a decrease in primary extinction.

Using the Darwin formula for the extinction correction, an approximate value of *D*, the extent of the originally coherent scattering blocks has been calculated. For the silver iodide samples this was in the vicinity of 10^−5^ cm. This is larger than for most crystals by about a factor of ten. It can be compared to an estimated average penetration of about 2×10^−5^ by X-rays into the powder slab, calculated from the published linear absorption coefficients for the silver and iodine atoms. Silver iodide thus presents a very unusual case, where linear absorption limits the penetration to the order of the perfect region.

The major deviation from the extinction behavior is shown by those reflections which can be indexed on the basis of both the hexagonal wurtzite-type and the face-centered cubic zincblende-type structures. For hexagonal indexing these are the (002) and (110) reflections. Neither of these two reflections changes appreciably during the early stages of this process indicating that the coherence of blocks contributing to these remains unchanged. Both structures consist of expanded close-packed layers of iodine atoms, with silver atoms between them. Only the stacking sequence of the layers differs for these two structures. Thus changes in the stacking sequence or displacement of silver atoms to interstitial sites would leave the intensity from these reflections invariant, although the interplanar spacing might not remain constant.

The second stage, indicated by an increase in the intensity of the (002) reflection, did not begin until the initial stage was substantially completed. No marked change was observed for the (110) reflection, probably because extinction effects are limited to reflections with large intensity and low Bragg angles.

The explanation for this behavior on the basis of the powder diffraction data was difficult. For this reason the effect of irradiation by light on large single crystals was further investigated. Laue photographs of a silver iodide crystal showed unambiguously that the second anomalous intensity change was due to fragmentation. This work will be described in the following section.

## 6. Single Crystal Laue Photographs

**Figure 4:**
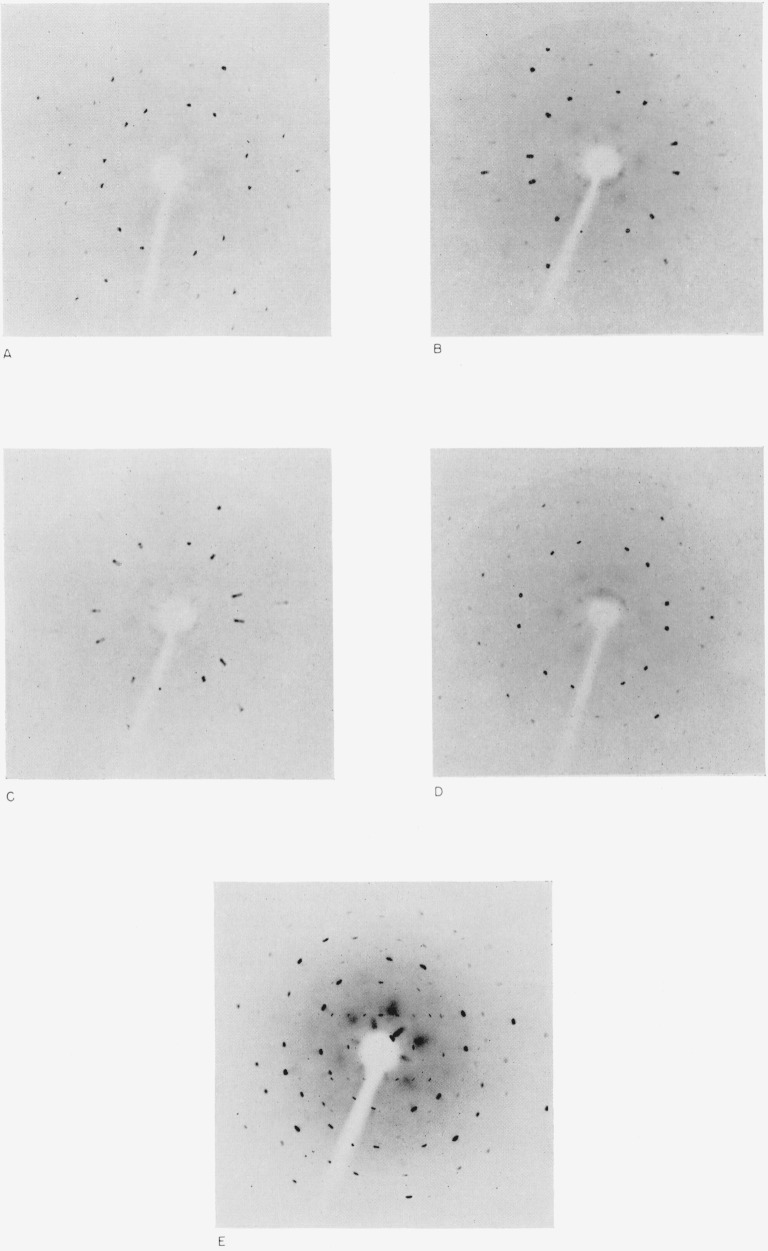
Transmission Laue X-ray diffraction patterns for a thin, single crystal of hexagonal silver iodide after various exposure times to light in the visible region. Initial (0) hr.20 hr.30 hr.70 hr.100 hr.The scale is reduced 2:1.

Since the conclusions reached from the extinction theory were largely inferential, an effort was made to obtain further evidence by other techniques. Transmission Laue X-ray diffraction photographs were obtained from a large single crystal after varying cumulative periods of exposure to the microscope lamp. The various stages of the observed changes in the single crystal patterns are shown in figure 4a–e. These were recorded at cumulative exposure times to light of 0, 20, 30, 70, and 100 hr, respectively.

The first picture of this series shows the pattern obtained before any irradiation with light. The diffraction maxima are irregular. This is characteristic of the type pattern obtained from a single perfect crystal. The next two pictures were made after 20 and 30 hr exposure time, respectively. These show a pronounced asterism, or radial streaking of diffraction spots, increasing with time. This can be readily explained only on the basis of a variability in the *c*/*a* axial ratio, such as would be expected to occur in internally strained crystals [6], or by a one-dimensional distortion of the lattice.

The next picture was made after a 70 hr exposure. The radial asterism has completely disappeared at this point and the diffraction spots are all approximately circular. This type of pattern is characteristic of an ideally mosaic crystal. The last picture shows the pattern after a 100 hr cumulative exposure time. This shows an elongation of the spots in a tangential direction, as well as a number of weak spots not previously observed. This is indicative of a fragmentation of the crystal into small crystallites, but with approximate retention of spatial orientation. As a limit, the tangential elongation into a complete circle would be obtained for a collection of crystallites with random orientation.

**Figure 5:**
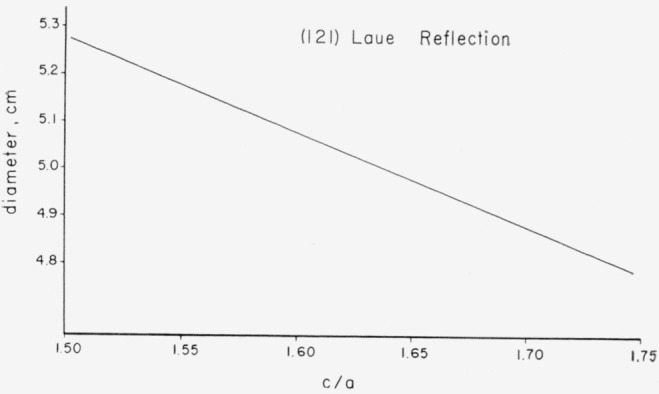
Calculated *c/a* axial ratios for the (121) Laue reflection as a function of the measured diameter.

The *c*/*a* ratio for the ideal wurtzite lattice is 1.633. The (121) Laue reflection was indexed and the spread of *c*/*a* ratios calculated. The details are given in figure 5. The maximum *c*/*a* ratio was 1.69 and the minimum was 1.59 just before an ideally mosaic crystal formed. This is almost certainly associated with the progressive decrease in the size of the coherently scattering blocks that occurred during the first stage of the change in the powder patterns.

## 7. Radiation Damage

The introduction of increased disorder in the structure by light is to some extent comparable to the general field of radiation damage. In the latter case very energetic particles or radiation give rise to localized defects. The atoms are displaced by collision, with a certain amount of the energy of the impinging particle transferred to an atom in the crystal. This can lead to secondary displacement of adjacent atoms. In general, fast neutrons are required to effect atomic displacements. Considerably more energy is required for this than is necessary to cause the migration of a silver atom to an interstitial site in silver iodide.

Two samples of silver iodide powder were exposed separately to different high energy radiation in order to determine the effect on the lattice. One was given a total dose of 1 roentgen by 250 kv X-rays, and the other 10 roentgens by Co^60^ *γ*-radiation. In neither case was there any change in the powder X-ray diffration pattern. This indicates that silver iodide is either not subject to visible damage by considerable doses of radiation from sources of this kind, or that the effect is random to such an extent that it does not appreciably affect the coherent scattering.

Even after extended periods of exposure to X-rays in the 50 kv range no visible darkening was observed. A preliminary quench in liquid nitrogen to reduce the perfection of the crystallites and subsequent exposure to X-rays of the same type produced slight darkening after times of less than 4 hr. This suggests that the silver iodide lattice is sensitized to ionizing radiation by the introduction of some disorder.

## 8. Mechanism

A qualitative explanation can now be given for the observed behavior of silver iodide in terms of the theory of the photographic latent image.

The latent image is believed to consist of submicroscopic specks of silver in some of the grains. Experimentally, it has been determined that the development of the latent image always begins at grain boundaries, slip planes, or impurity centers [7]. The introduction of strain into the lattice produces many more nucleation centers than exist in the equilibrium structure.

The first satisfactory theory of the latent image was proposed by Gurney and Mott in 1938 [8]. There is evidence that the primary absorption in the AgX crystal occurs as

$$X^{-}+hv\to X^{0}+e^{-}.$$

Light absorption in the ultraviolet region is required to excite an electron from an X^−^ ion in an ordinary lattice position. The absorption at longer wavelengths is probably due to X^−^ ions at dislocations or other disturbed positions in the structure.

Since AgI is a semiconductor the quantum mechanical concepts of the band theory can be used. The electron is raised from a valence to a conduction band where it can move freely. Hence the AgI becomes photoconducting. The mobile electron moves through the crystal until it becomes trapped by a pre-existing silver nucleus or in a low-lying level of a sensitizer. Thus a center of negative charge is produced, attracting Ag^+^ atoms which can move through the crystal via interstitial positions. When a Ag^+^ ion reaches the negative center it is neutralized

$${\text{Ag}}^{+}+e^{-}\to {\text{Ag}}^{0}.$$

The silver nucleus is thus enlarged and continues to grow by the same mechanism.

The neutral X atom eventually reaches the surface by a process of electron transfer

$$X^{-}{\text{+X}}^{0}\to X^{0}{\text{+X}}^{-}.$$

The X atom is a center of missing negative charge and is called a positive hole. The theory required that these positive holes not recombine with the electrons.

In 1953 Hedges and Mitchell modified this theory slightly on the basis of extensive experiments [9]. They proposed that the function of the sensitizer is to trap positive holes, preventing their recombination with electrons. The electrons would then be trapped at crystal imperfections, and the silver particles grow there to form the latent image.

Silver iodide undergoes a photolytic reaction similar to the other silver halides, but at a considerably slower rate. This is in agreement with some very recent work by James and Vanselow [10, 11] on the kinetics of the development of silver iodide emulsions. Neutral silver atoms are formed by combination of electrons and silver ions. The rate of diffusion of these silver ions through the lattice is relatively much more rapid than that of iodine atoms. The migration path is by way of interstitial positions. The change in size and bonding of the silver atoms is sufficient to lead to a lattice distortion. The neutral silver atoms tend to move toward existing defect sites. There they may be effectively trapped and immobilized. The existence of aggregates of these silver atoms at grain boundaries has been shown by Mitchell [12–14] for silver chloride. A similar movement of silver atoms in the silver iodide structure is very probable. The high mobility of the silver with respect to the iodine atoms in silver iodide is well substantiated. The collection of a number of the silver atoms at one dislocation site may be sufficient to cause local plastic deformation. A large number of these small lattice misorientations would be sufficient to explain all the reported observations.

The elastic deformation limits of an ideal crystal are generally exceeded when the lattice change is greater than about 5 percent. At this point the small number of misoriented blocks can exert a strain sufficient to fragment the crystal into an essentially ideal mosaic structure. This stage is represented by the first plateau stage of the X-ray powder diffraction patterns.

Further exposure to light continues the process of misorientation of adjacent blocks due to the strains produced by further silver precipitation. This can continue until a collection of crystallites with substantial orientation differences is formed. The layer type structure appears to be generally preserved at this stage, leading to an enhancement of those reflections for which *h—k = 3N.* Simultaneously, there may occur an annealing of growth faults in the face-centered cubic lattice. The total effect is to maintain the remaining reflections essentially constant while the (002) and (103) reflections increase in intensity. This stage is represented by the latter portion of the powder diffraction pattern and the final Laue photograph.

The observed behavior of silver iodide thus appears to be analogous to that of the other silver halides insofar as the photolytic mechanism is concerned. The fundamental difference lies in the rate at which the reactions occur. The considerably slower rate for silver iodide has permitted a more-detailed mechanism to be observed for this compound which is in good agreement with the previously proposed theories of the photographic process.

## Figures and Tables

**Table 1:** Observed values of extinction coefficient for silver iodide

*hkl*	λ	*I_final_*	*I_init_*	*I_i_/I_f_*	*p*	*I_i_*/*pI_f_*	*ϵ*
							
100	Mo	56	35	0. 62	0. 99	0. 62	0.38
	Cu	72	40	.56	.93	.60	.40
	Fe	87	46	.54	.88	.61	.39
101	Mo	41	11	.27	.97	.28	.72
	Cu	48	12	.25	.90	.28	.72
	Fe	68	16	.23	.86	.28	.72
102	Mo	36	6	.17	.96	.18	.82
	Cu	42	6	.14	.85	.17	.83
	Fe	61	8	.13	.77	.17	.83
103	Mo	120	12	.10	.94	.11	.89
	Cu	108	10	.09	.76	. 12	.88
	Fe	150	12	.08	.67	.12	.88

**Table 2:** Calculated values of extinction coefficients for silver iodide

λ	*hkl*	*θ*	*F^2^_rel_*	*d*_2_×10^16^	*A*/γ_0_γ_*r*_	*ϵ*_rel_
						
Fe	100	14.07	21	15.86	1.26	0. 36
	101	16.00	38	12. 33	1.80	.73
	102	20.76	35	7. 46	3.70	.83
	103	27.18	100	4.49	2. 30	.89
Cu	100	11.15	21	15. 86	1.04	.35
	101	12.67	38	12. 33	1.50	.70
	102	16.38	35	7. 46	3.13	.82
	103	21.32	100	4.49	2.00	.89
Mo	100	5.11	21	15. 86	1.00	.39
	101	5.80	38	12. 33	1. 31	.73
	102	7.46	35	7.46	2.63	.81
	103	9.63	100	4. 49	1.65	.88

## Footnotes

3 Figures in brackets indicate the literature references at the end of this paper.
